# Improving Communication With Relatives on an Older Adult Mental Health Inpatient Ward: A Quality Improvement Project

**DOI:** 10.7759/cureus.114159

**Published:** 2026-08-08

**Authors:** Oliver Tran, Christianna Ramsundar, Ekaterina Doukova

**Affiliations:** 1 Psychiatry, North London NHS Foundation Trust, London, GBR

**Keywords:** communication difficulties, communication in healthcare, covid-19, effective communication, geriatric psychiatry, inpatient care, psychiatric inpatient care, quality improvement projects, verbal communication, written and verbal communication

## Abstract

Introduction

Communication with relatives is a vital part of delivering high-quality healthcare, particularly within older adult mental health inpatient services where many patients have cognitive impairment or severe mental illness. The involvement of family members is especially crucial given that patients often rely upon family members to support decision-making and discharge planning. During the COVID-19 pandemic, visiting restrictions drastically reduced opportunities for face-to-face communication, resulting in increased anxiety amongst relatives and increased telephone enquiries to ward staff. This quality improvement project (QIP) aimed to improve the quality, consistency and frequency of communication with relatives of patients admitted to an older adult psychiatric inpatient ward by introducing a structured written communication process, with the goal of increasing relatives' involvement in care and reducing telephone requests for clinical updates.

Methods

A QIP was undertaken on a 20-bed older adult psychiatric assessment ward within North London NHS Foundation Trust to improve the quality, consistency and frequency of communication with relatives. Baseline feedback from relatives identified a wish for more regular, structured updates, with a majority expressing a preference for written communication via email. Four iterative Plan-Do-Study-Act (PDSA) cycles were undertaken to develop and refine a standardised written feedback template that was emailed to relatives following multidisciplinary ward reviews.

Results

Satisfaction amongst relatives improved throughout the project, with mean feedback scores increasing from 4.25/5 during the first cycle to 4.94/5 by the fourth and final cycle. Relatives consistently reported feeling better informed and more involved in their family member's care. Nursing and administrative staff also reported fewer telephone enquiries requesting clinical updates, suggesting reduced administrative burden and improved efficiency.

Conclusions

The intervention proved acceptable to relatives and staff, required minimal financial investment, and became embedded into routine clinical practice. Standardised written communication represents a simple, sustainable intervention that may improve family experience and reduce staff workload within older adult mental health inpatient services.

## Introduction

The Oaks is a 20-bed acute inpatient mental health ward located at Chase Farm Hospital in Enfield, London. It provides assessment and treatment of older adults (over the age of 65 years) suffering from both functional and organic psychiatric disorders. This includes mental health problems such as mild to moderate dementia, depression and psychotic illnesses that cannot be treated and supported appropriately at home or in an alternative setting [1]. A multidisciplinary team (MDT) of psychiatrists, nurses, psychologists, occupational therapists, physiotherapists, healthcare assistants, etc. support the patients on the ward. The ward supports the catchment area of North London, home to an ethnically diverse [2] and ageing population [3]. The Oaks is part of the North London NHS Foundation Trust, which provides services across five boroughs in North London [4].

Communication with relatives is a vital part of delivering person-centred healthcare [5]. Family members are involved in every stage of a patient’s care, ranging from providing collateral history to supporting with treatment decisions to advising a safe and effective discharge plan [6]. This is especially important for older adult patients, who may suffer from conditions (including dementia [7]) that affect their capacity to make decisions regarding their own care. Relatives of patients admitted to The Oaks reported that difficulties in receiving regular updates or visiting the ward made it difficult for them to hear about changes to treatment, clinical progress and discharge planning.

The COVID-19 pandemic exacerbated these challenges by further restricting the opportunities for relatives to visit the ward and speak to staff in-person, thereby increasing the reliance on telephone updates [8]. However, because of varying shift patterns, high clinical workload and the dynamic nature of an acute psychiatric ward, information provided by different members of staff often varied in consistency and detail. Relatives were reported to prefer updates from medical staff, which resulted in repeated telephone calls and interruptions to clinical work.

The aim of this project was to explore concerns raised by relatives regarding communication and then to improve the quality and frequency of communication shared with relatives.

The objectives were: (1) to improve the quality and frequency of communication with relatives; (2) to standardise communication with relatives; (3) to improve the involvement and understanding of relatives in the care of their family member on the ward; and (4) to reduce avoidable telephone requests for clinical updates from relatives by providing proactive, structured communication, thereby allowing clinicians to spend more time delivering direct patient care.

Background

Effective communication, defined as the timely, accurate, consistent and understandable exchange of information that enables patients and their families to participate in decision making [5,9], is a fundamental component of high-quality, person-centred healthcare. In mental health services, relatives can face uncertainty regarding the diagnosis, treatment plan and discharge arrangements, particularly when patients lack capacity to make these decisions or have cognitive impairment [10].

National guidance from the General Medical Council (GMC) [5] and the National Institute for Health and Care Excellence (NICE) [9] both stress the importance of involving relatives and carers in patient care, whilst also respecting the patient’s wishes and their right to confidentiality. The GMC advises that doctors should communicate appropriately with those close to the patient whilst respecting the patient's wishes and right to confidentiality. Where possible, consent should be sought before sharing information. However, if a patient lacks capacity, doctors may share relevant information with family members or those close to the patient where this is in the patient's best interests, and there is no reason to believe that the patient would have objected [5].

This is supported by studies which consistently demonstrate that improved communication enhances family satisfaction, reduces anxiety and supports shared decision-making. A study by Wittenberg et al. in 2020 [11] showed that caregivers who experienced communication difficulties reported higher anxiety and poorer quality of life, whereas greater communication and self-efficacy were associated with lower anxiety and better quality of life. A systematic review by Seif et al. in 2022 [10] uncovered that despite robust policies intended to improve inpatient experiences, relatives and carers have particularly negative experiences and largely report being dissatisfied with how they and their loved one are treated during inpatient care. Finally, a UK focus group study in 2017, through thematic analysis, found that carer involvement should happen as soon as possible after admission and, by doing so, personal knowledge of the patient can be utilised whilst highlighting the challenges due to pinpointing carers during a crisis, obtaining relevant patient consent and contacting identified persons [6].

The COVID-19 pandemic had wide-ranging impacts on hospital service provision, including communication between healthcare staff and relatives [8,12]. Restrictions on face-to-face visits meant that telephone and digital communication methods [13] were adopted to maintain contact [14], but these interventions often lacked standardisation and depended heavily on individual clinicians. Within The Oaks, communication was largely reactive, providing relatives with updates whenever they called the ward. However, this approach resulted in variability in the detail and accuracy of the information that was shared and caused disruption to clinicians who were delivering patient care in an acute and dynamic environment.

The quality improvement project (QIP) therefore sought to establish a structured, proactive communication process that could be integrated into routine ward practice.

## Materials and methods

Measurement

The QIP was conducted on The Oaks older adult mental health inpatient ward over a four-week period in March 2022. The QIP's primary outcome measure was the patient relatives’ satisfaction with their experience of communication with the ward. This was because the principal aim was to improve the relatives’ experience and involvement in the care of their loved ones. Satisfaction amongst patient relatives was measured using both quantitative and qualitative measures.

Prior to implementation of the feedback template, baseline satisfaction was assessed using structured qualitative questionnaires that were prepared and distributed to a simple random sample of five relatives of current inpatients (see Appendices). Eligible participants included adult relatives or next of kin who had regular contact with the patient and were involved in communication regarding their care. Relatives who were unavailable to participate, declined participation, or were not identified as an appropriate contact for communication were excluded. Relatives were invited to participate voluntarily in the baseline questionnaire and were informed that their feedback would be used to evaluate and improve communication on the ward. Patients who did not have relatives were also excluded. Completion of the questionnaire was taken as implied consent to participate. Communication with relatives occurred only within the context of routine clinical care and in accordance with local Trust policies regarding patient confidentiality and consent. The questions explored their thoughts on the QIP, consent to participate, experience of the existing system of communication and updates, satisfaction with the existing system, preferred frequency of communication, preferred method of communication, as well as space for other comments and suggestions. This feedback was then reviewed and summarised to inform the intervention that was applied.

Following implementation of the intervention, relatives receiving written feedback were invited to rate the quality of the communication using a five-point Likert scale (1 = poor, 5 = excellent) and provide qualitative comments regarding the usefulness and clarity of the information received. Satisfaction scores were reviewed after each Plan-Do-Study-Act (PDSA) cycle to identify trends over time. A follow-up telephone questionnaire was undertaken during the final PDSA cycle to explore whether relatives felt more informed, more involved in care, and whether the frequency and content of the updates were appropriate. The use of both quantitative ratings and qualitative feedback improved the validity of the evaluation by allowing numerical trends to be interpreted alongside relatives' experiences [15,16].

Besides outcome measures, process measures were also assessed to see whether the intervention was delivered consistently. This included the number of written feedback forms completed and emailed each week, the frequency of communication (which changed from weekly to fortnightly) and the time taken to complete each feedback form. Throughout the PDSA cycles, the communication template was refined to improve efficiency. During PDSA cycle 1, clinicians reported that completion of the initial communication template required approximately 15-20 minutes per new form. Following modifications made after PDSA cycle 1, including removal of duplicated sections, completion time for a new form reduced to approximately 10-15 minutes, while updating an existing form required only 5-10 minutes. In PDSA cycle 2, the ward administrator also advised that eight forms a week represented the maximum sustainable workload, which informed subsequent implementation.

Finally, balancing measures were used to assess whether the intervention generated unintended consequences elsewhere within the service. As one of the project objectives was to reduce telephone enquiries from relatives requesting updates, informal feedback was collected from nursing and administrative staff regarding changes in the frequency of these calls. Staff were asked whether the intervention affected their workload or the delivery of patient care. Although no objective call log data were collected, nursing and administrative staff consistently reported fewer requests for updates from relatives and fewer requests for doctors to return telephone calls following implementation of the written communication system. Staff also considered the intervention to be sustainable once the communication template had been simplified.

Design

This QIP was designed and led by two resident doctors, Dr Dilyana Andonova and Dr Jade Wright, with supervision from the consultant psychiatrist at The Oaks, Dr Ekaterina Doukova. Ward nursing and administrator staff also supported the project by relaying feedback from relatives on the intervention. As this was a QIP and not a research study, it met the Trust's local quality improvement approval process and was exempt from needing ethics committee approval. A baseline questionnaire, also developed by Dr Dilyana Andonova, Dr Jade Wright and Dr Ekaterina Doukova, was prepared to obtain feedback on the relatives’ experience of the existing process of communication with the ward. The baseline and follow-up questionnaires were developed by the project team to evaluate relatives' experiences of communication with the ward and to inform iterative refinement of the intervention throughout the PDSA cycles.

Following analysis of baseline questionnaires, the team designed a standardised written feedback template to be distributed to patients’ relatives on a regular basis. The communication update template contained information on the patient’s diagnosis, relevant medications and recent changes, feedback from respective members of the MDT, the patient’s own reflections, the current treatment plan and discharge plans. The forms were stored on the ward’s Shared Drive and sent to relatives on a predetermined date.

**Figure 1:**
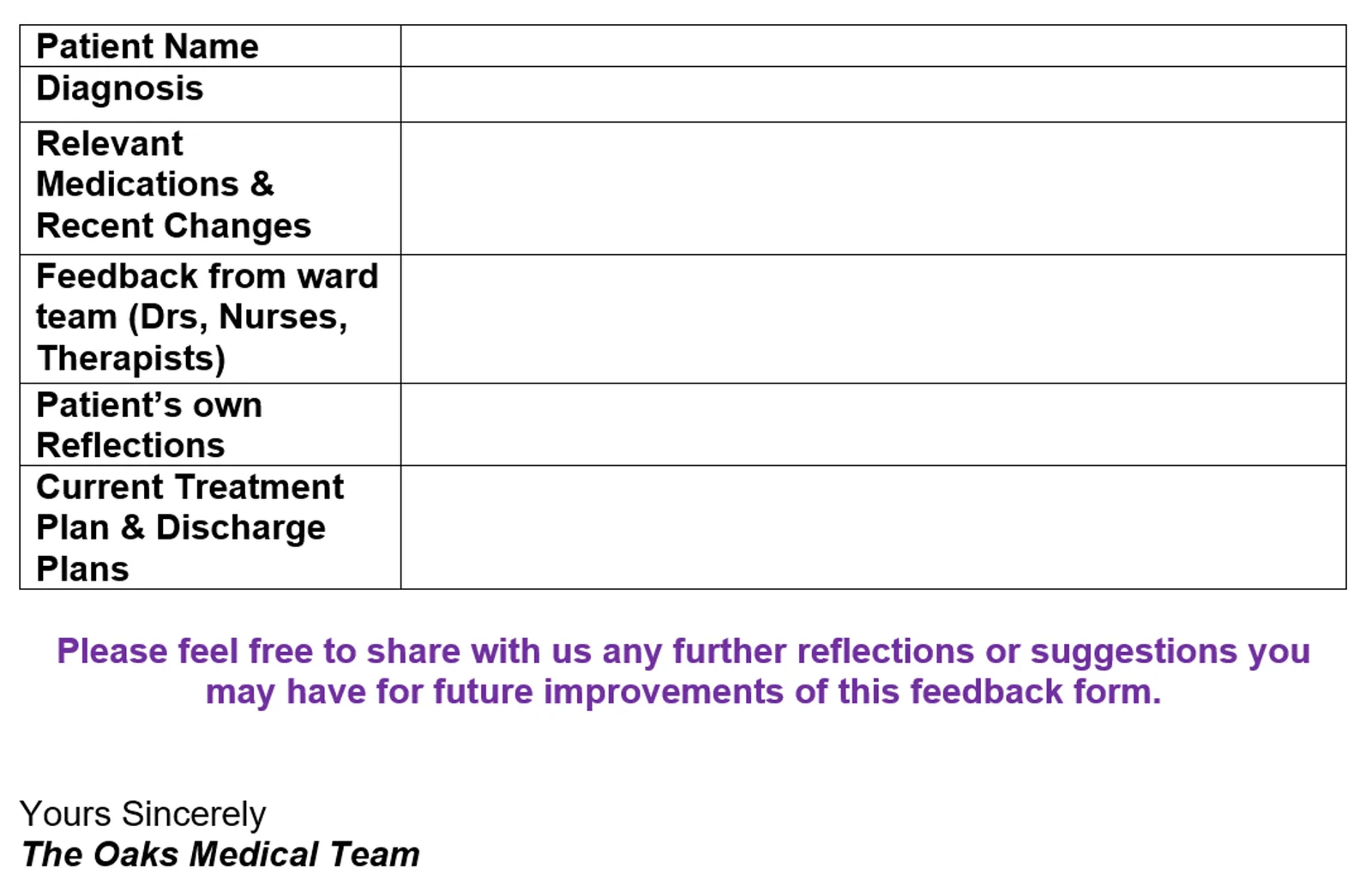
Template used for providing family communication updates after PDSA cycle 1. PDSA: Plan Do Study Act

Initially, separate sections were included for medical, nursing and therapy updates. However, following the implementation of the first PDSA cycle, these were condensed into a single multidisciplinary summary to reduce duplication of work and the time taken for staff to complete the template (Figure 1). The ward administrator supported this by sending the completed forms to relatives by secure email.

Strategy

**Figure 2:**
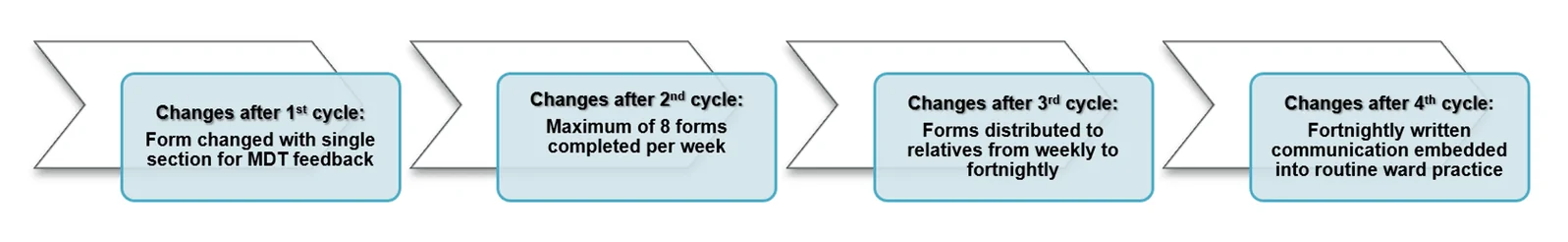
Summary of changes made with each Plan-Do-Study-Act (PDSA) cycle. MDT: multidisciplinary team

The SMART [17] aim was to improve the quality, consistency and frequency of communication with relatives by introducing a structured template form to provide feedback about their family member on the ward. Four PDSA test cycles were undertaken over four weeks in March 2022 (Figure 2). A pragmatic needs-based sampling approach was used throughout the PDSA cycles. Relatives of patients requiring ongoing inpatient assessment and treatment, for whom regular communication was clinically appropriate, were invited to participate. As the intervention was refined, some relatives continued to receive communication across successive PDSA cycles, while additional eligible relatives were included to evaluate subsequent iterations of the communication template.

PDSA Cycle 1

A draft written communication template was introduced to a sample of five relatives of current inpatients requiring ongoing clinical updates. Following distribution of the template, structured feedback was obtained from relatives using a five-point Likert scale and free-text comments. Clinicians also provided feedback regarding the usability of the template, which informed subsequent modifications.

PDSA Cycle 2

The revised communication template was implemented with three additional relatives, while the original participants continued to receive communication updates. Feedback was again collected from relatives and clinicians to assess the acceptability, feasibility and practicality of the revised template.

PDSA Cycle 3

The communication process was further refined by modifying the frequency of written updates from weekly to fortnightly in order to improve sustainability and increase the number of families able to receive updates. Some relatives continued from the previous cycle, while three additional eligible relatives were included. Relative feedback continued to be collected following implementation.

PDSA Cycle 4

The final version of the communication template was implemented and evaluated using follow-up questionnaires with five relatives of patients who remained on the ward and required ongoing clinical care necessitating regular communication with their families. All qualified ward staff were also provided the opportunity to provide feedback, as well as the ward administrator, given their interaction and interface with relatives. The nursing team provided general feedback by reflecting on the QIP's impact on the number of calls they had received from relatives. Following completion of the fourth cycle, the intervention was considered for incorporation into routine ward practice.

Data analysis

Quantitative data were analysed using descriptive statistics. Mean satisfaction scores were calculated following each PDSA cycle to assess changes in relatives' satisfaction over time. Given the quality improvement design and small sample size, no inferential statistical analyses were performed. Microsoft Excel (Microsoft Corp., Redmond, WA, USA) was used to calculate descriptive statistics and present the results.

Qualitative feedback from relatives and ward staff was reviewed descriptively and grouped into recurring themes relating to communication quality, involvement in care, usability of the communication template and operational impact. These themes were used to inform iterative refinements during successive PDSA cycles. Recurring comments regarding communication quality, usability of the feedback template and perceived impact on clinical workflow were identified and used to inform iterative modifications during successive PDSA cycles. A formal qualitative analytical framework, such as thematic analysis, was not undertaken because the primary purpose of collecting qualitative feedback was to support real-time service improvement rather than qualitative research.

## Results

**Figure 3:**
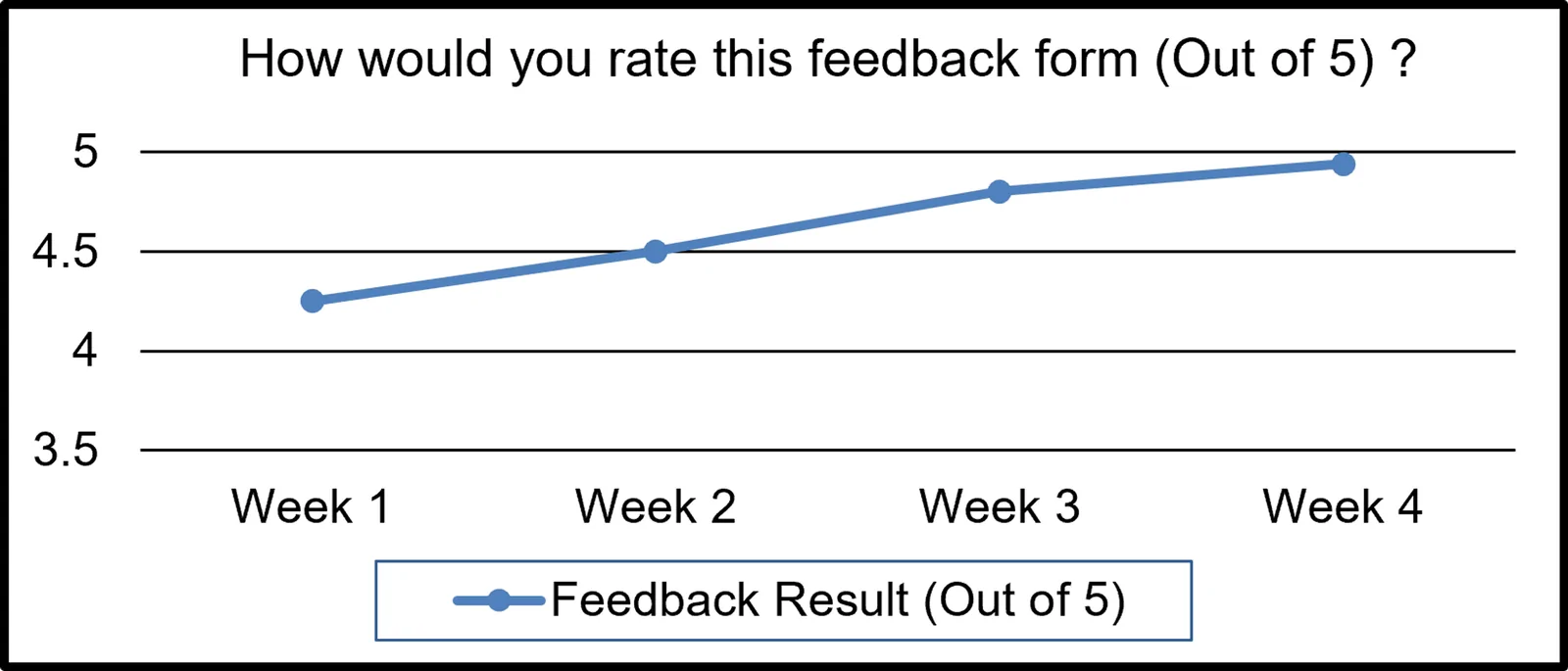
Average relative satisfaction scores over successive PDSA cycles. PDSA: Plan Do Study Act

The intervention demonstrated an incremental improvement throughout successive PDSA cycles (Figure 3). Average relative satisfaction scores progressively improved from 4.25 (Week 1) to 4.5 (Week 2) to 4.8 (Week 3) to 4.94 (Week 4).

PDSA cycle 1

Five relatives received the initial communication template on 03/03/2022. All five relatives responded, with a mean satisfaction score of 4.25/5. Although relatives reported positive experiences with the structured communication, clinicians considered the template unnecessarily detailed and unnecessarily time-consuming, requiring approximately 15-20 minutes to complete. Consequently, the template was simplified by removing duplicated sections and combining the medical, nursing and therapy sections into a single MDT summary.

PDSA cycle 2

The revised template was distributed to three additional relatives on 10/03/2022, resulting in a total of eight participants. All eight relatives responded, and mean satisfaction increased to 4.5/5, supported by positive qualitative feedback from relatives. Following simplification of the template, clinicians reported that completion time for a new form reduced to approximately 10-15 minutes, while updating an existing form required only 5-10 minutes. The ward administrator considered completion of eight templates per week to represent the maximum sustainable workload.

PDSA cycle 3

Three relatives from the previous cycle continued to receive updates, and three additional relatives were included, giving a total of six participants who were sent updates on 17/03/2022. Following implementation of fortnightly updates, the mean satisfaction score increased to 4.8/5. Although only three relatives returned formal feedback, comments continued to be highly positive, with one relative describing the team as compassionate, helpful and reassuring.

PDSA cycle 4

Five relatives received written communication during the final PDSA cycle on 24/03/2022. These were selected from a list of patients who were provided ongoing care and necessitated updates to be provided to their families, rather than those awaiting discharge for social issues. All five relatives responded, and mean satisfaction further increased to 4.94/5. Follow-up telephone questionnaires indicated that relatives felt better informed and more involved in their family member's care. Nursing and administrative staff also reported fewer telephone requests for clinical updates and fewer requests for doctors to return calls. Following completion of the project, fortnightly written communication was embedded into routine ward practice.

The progression of the intervention and associated operational outcomes across the four PDSA cycles is summarised in Table 1. Relative satisfaction improved progressively following each cycle, while iterative refinements reduced clinician workload and improved the sustainability of the intervention. Completion time for a new form reduced from approximately 15-20 minutes during PDSA cycle 1, to 10-15 minutes following refinement of the template in PDSA cycle 2, while updating an existing form required only 5-10 minutes. Reducing the frequency of updates to fortnightly during PDSA cycle 3 further improved sustainability, and by the final cycle, nursing and administrative staff reported fewer requests for clinical updates and fewer requests for doctors to return calls.

**Table 1:** Summary of intervention modifications, process measures and outcomes across the four PDSA cycles Completion time for the initial template (15-20 minutes) was estimated from clinician feedback during PDSA cycle 1. Formal evaluation of completion times following template refinement was undertaken during PDSA cycle 2 and not repeated thereafter. PDSA: Plan Do Study Act; MDT: multidisciplinary team

PDSA cycle	Relatives receiving updates (n)	Responses (n)	Mean relative satisfaction (out of 5)	Update frequency	Time to complete new form	Time to update existing form	Major modifications	Key findings
1	5	5	4.25	Weekly	15-20 mins	-	Simplified template by merging MDT sections	Positive relative feedback; clinicians found template too detailed
2	8	8	4.5	Weekly	10-15 mins	5-10 mins	Revised template implemented	Improved satisfaction; workload considered sustainable at eight forms per week
3	6	3	4.8	Fortnightly	-	-	Reduced update frequency	Satisfaction maintained with improved sustainability
4	5	5	4.94	Fortnightly	-	-	Final template embedded	Reduced telephone requests; intervention adopted into routine practice

Quantitative data was reinforced by qualitative feedback, which consistently highlighted an improved understanding of treatment plans, reassurance regarding patient care, and appreciation of receiving consistent information from the MDT. Representative comments from patient relatives included “*This has been an excellent summary of what was discussed at the meeting and greatly appreciated*” and “*The level of service has been outstanding and has exceed my highest expectations*”. Relatives were satisfied with the detail of the feedback, commenting that the form was "*perfect*", "*got everything*", and "*comprehensive enough*".

Likewise, feedback received from ward staff was also positive, reporting operational benefits in reducing workload. Representative comments from admin staff include: “*All feedback has been positive…next of kin are very grateful to hear about/understand the treatment plan that is in place for their family member and also have an update of their condition*”. Nursing staff described spending less time responding to telephone enquiries from families requesting updates on their loved ones, and administrative staff noted fewer requests for doctors to telephone relatives. A notable point of reflection was the frequency of updates, as fortnightly updates may be appropriate for longer stay patients, whereas patients who were more recently admitted may need more frequent updates.

Qualitative feedback from relatives and ward staff identified several recurring themes relating to the quality of communication, involvement in care, usability of the communication template and sustainability of the intervention, summarised in Table 2.

**Table 2:** Summary of qualitative feedback themes and its impact on intervention refinement MDT: multidisciplinary team

Theme	Representative feedback	Impact on intervention
Communication was valued and reassuring	"*This has been an excellent summary...greatly appreciated*."	Reinforced continuation of structured written communication.
Relatives felt more informed and involved in care	During follow-up interviews, all relatives reported feeling more informed and involved in their family member's care, describing the intervention as making a "*huge difference*".	Supported continuation of regular written updates as part of routine care.
Communication template was comprehensive and easy to understand	Relatives described the form as "*perfect*", "*got everything*", and "*comprehensive enough*".	No substantial changes to the content of the final template were required.
Need to improve usability and sustainability	Clinicians reported that the initial template contained unnecessary duplication and was time-consuming to complete.	Medical, nursing and therapy sections were merged into a single MDT section, simplifying completion.
Fortnightly communication was acceptable for most patients	Relatives generally considered fortnightly communication sufficient, although some suggested weekly updates for newly admitted patients.	Update frequency changed from weekly to fortnightly, while recognising newly admitted patients may require more frequent contact.
Reduced administrative burden	Nursing and administrative staff reported fewer telephone requests for updates and fewer requests for doctors to return calls following implementation.	Intervention adopted into routine ward practice with continued fortnightly written communication.

## Discussion

Principal findings

Communication with relatives is an essential part of high-quality, person-centred care within older adult mental health services, where family members frequently provide collateral information, contribute to decision-making and support patients throughout their admission and discharge [5,9]. This QIP demonstrated that introducing regular and structured written communication was associated with improved relative satisfaction, greater perceived involvement in care, and more consistent communication between the MDT and patients’ families.

The QIP achieved its primary aim of improving the quality, frequency and standardisation of communication with relatives by introducing regular written updates. The intervention also met its key objectives by improving relatives' understanding of their family member's care, increasing their perceived involvement in treatment, and establishing a more consistent approach to communication across the MDT. Successive PDSA cycles enabled the intervention to be refined in response to feedback from relatives and ward staff, resulting in a communication template that balanced comprehensive clinical information with the practical constraints of a busy inpatient ward. Mean relative satisfaction scores improved progressively throughout the project, while qualitative feedback found that families felt more reassured and more involved in the care of their loved ones.

Comparison with previous literature

The findings of this QIP are consistent with previous studies demonstrating that proactive and structured communication with relatives improves family experience within inpatient healthcare settings. Relatives who received regular written updates throughout this project reported progressively higher satisfaction scores and described feeling more informed and involved in their family member's care. Similarly, Giacco et al. found that relatives of psychiatric inpatients valued regular communication and involvement in care planning, highlighting communication as a key determinant of carer satisfaction and therapeutic relationships [6]. Likewise, Wittenberg et al. reported that better communication between healthcare professionals and family caregivers was associated with lower caregiver anxiety and improved quality of life, emphasising the importance of timely, understandable and supportive communication [11].

The iterative refinement of the communication template through successive PDSA cycles is also consistent with quality improvement methodology described by Langley et al. and Ogrinc et al., who emphasise the importance of repeated testing, measurement and stakeholder feedback when developing sustainable service improvements [15,16]. Feedback from both relatives and clinicians enabled the intervention to be simplified without compromising the quality of information provided, improving its practicality within a busy inpatient environment.

In addition to improving relatives' experience, ward staff perceived fewer telephone requests for clinical updates following implementation of the intervention. Although objective workload measures were not collected, this finding is consistent with recommendations published during the COVID-19 pandemic advocating proactive communication with relatives to reduce uncertainty and maintain family-centred care despite visiting restrictions [8,14]. The present QIP extends these recommendations by demonstrating that a structured written communication process can be integrated into routine practice within an older adult mental health inpatient ward without requiring additional staffing or resources.

Lessons learnt and sustainability

This QIP showed that making relatively simple changes to communication processes can have a meaningful impact on relatives' experience of inpatient mental healthcare. One of the key lessons learnt was the importance of engaging stakeholders throughout the improvement process. Baseline questionnaires allowed relatives to identify their preferred method and frequency of communication before the intervention was designed, ensuring that the changes addressed their needs. Similarly, feedback from clinicians, nursing staff and the ward administrator throughout successive PDSA cycles enabled the communication template to be refined, improving both its usability and sustainability.

The iterative nature of the PDSA methodology was key to the success of the project [15,16]. Feedback from clinicians completing the communication templates indicated that the initial version was overly detailed and time-consuming to complete. Through repeated testing and refinement, unnecessary duplication was removed, and multidisciplinary information was consolidated into a single section, substantially reducing the time required to complete each form while maintaining the quality of information provided. This highlights the value of continuous measurement and adaptation when implementing quality improvement interventions.

Another important lesson was that sustainability should be considered from the outset of a QIP. The involvement of the ward administrator in distributing the communication forms by secure email and storing them within the electronic patient record allowed the intervention to become integrated into existing ward processes, rather than relying solely on individual clinicians, who are often temporarily placed on the ward for six-month rotations. Following successful completion of the QIP, fortnightly written communication was incorporated into the routine information provided to relatives on admission and continued as part of standard ward practice until November 2024, when it was paused because of staffing pressures and changes to the ward structure. These findings suggest that successful implementation depended not only on the design of the intervention itself, but also on organisational stability, administrative support and sufficient staffing to maintain the process over time.

Operational impact

Besides improving the experience of relatives, the QIP demonstrated potential operational benefits. Nursing and administrative staff reported fewer telephone requests for clinical updates and fewer requests for doctors to return calls, suggesting that proactive communication can reduce interruptions to clinical work and allow doctors to spend more time with direct patient care. Although these observations were based on staff feedback rather than objective workload data, they indicate that structured communication may improve both the quality and efficiency of care.

Limitations

Despite these strengths, the project has several limitations. It was conducted on a single 20-bed older adult psychiatric inpatient ward, limiting the generalisability of the findings to other clinical settings. The number of relatives who participated was relatively small, and not all relatives returned feedback following each PDSA cycle, increasing the possibility of response bias. Those who chose to provide feedback may have been more engaged or more satisfied with the intervention than those who did not respond.

The evaluation relied predominantly on descriptive data, including satisfaction scores and qualitative feedback. While these measures demonstrated consistently positive trends, no validated communication or carer satisfaction questionnaire was used, and no statistical analysis was performed because of the small sample size. Furthermore, although nursing and administrative staff consistently reported a reduction in telephone enquiries from relatives following implementation of the intervention, objective data such as telephone call logs were not collected. Therefore, reductions in staff workload cannot be quantified and should be interpreted cautiously.

The project was also undertaken during the COVID-19 pandemic, when visiting restrictions substantially changed communication between healthcare professionals and relatives. Although the intervention addressed a need that was apparent during this period, the degree of benefit may differ if the project was conducted after visiting restrictions were relaxed. Nevertheless, proactive written communication may still be valuable by improving consistency of information, supporting carers who are unable to attend the ward regularly, and reducing unnecessary interruptions to clinical staff.

Patient, relative and staff demographic characteristics were not collected because the project evaluated a service-level communication process, rather than patient-specific outcomes. Consequently, it was not possible to explore whether the intervention performed differently across demographic or diagnostic subgroups.

Future implications

This project adds to existing evidence [6,10,11] that structured, proactive communication can improve the experience of relatives within inpatient settings. Although undertaken in a single older adult mental health ward, the intervention is likely to be transferable to other psychiatric inpatient services and acute hospital wards where effective communication with relatives is fundamental to delivering person-centred care. The findings also support the use of structured, proactive communication with relatives into local ward procedures and routine multidisciplinary practice, particularly in inpatient settings where relatives play an important role in providing collateral information and supporting shared decision-making.

Future quality improvement work should seek to evaluate the intervention across multiple inpatient wards using larger sample sizes and objective measures of communication efficiency, including telephone call frequency, clinician time, and validated measures of relative satisfaction. Longer-term evaluation would also help determine whether improvements are sustained over time and whether enhanced communication influences wider outcomes such as discharge planning, carer burden or patient experience. This approach could inform local communication policies and standard operating procedures for inpatient mental health services, if similar benefits are demonstrated in larger, multicentre QIPs. Future work should also evaluate this approach across multiple sites using objective workload measures and validated outcome instruments to further establish its effectiveness and wider applicability.

## Conclusions

The intervention required no additional funding, staffing or specialist technology, making it a low-cost and readily implementable quality improvement initiative. It was successfully integrated into routine ward practice during the project period, demonstrating that structured communication with relatives can be sustainably incorporated into existing clinical workflows within older adult mental health services.

Communication with relatives is an essential part of high-quality, person-centred care within older adult mental health services. This QIP demonstrated that introducing a structured written communication system was associated with improved relative satisfaction, greater involvement of relatives in patient care, and more consistent communication between the MDT and families. The intervention was inexpensive, sustainable and readily integrated into routine clinical practice, suggesting that similar approaches could be implemented across other inpatient mental health and hospital settings.

## Figures and Tables

**Figure 4:**
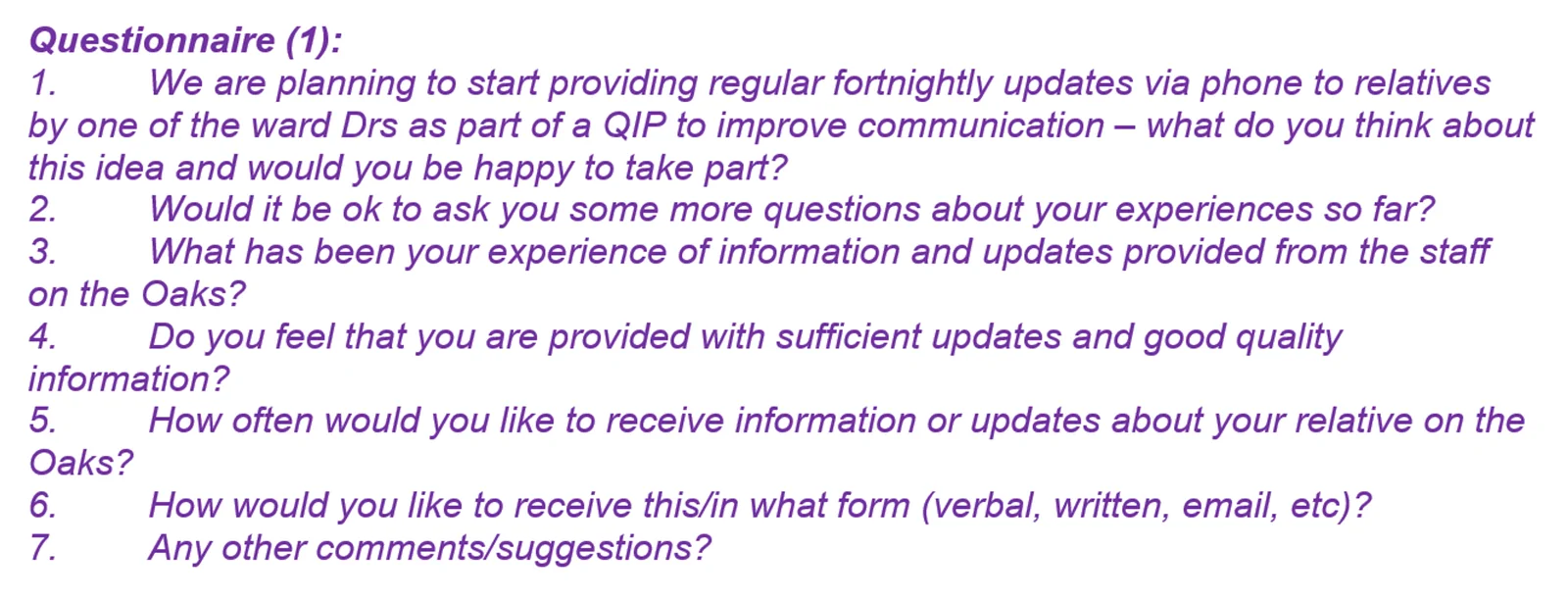
Questionnaire used to collect feedback from relatives regarding their experience of communication with the ward This was developed by two resident doctors on The Oaks, Dr Dilyana Andonova and Dr Jade Wright, as well as the ward consultant, Dr Ekaterina Doukova.

## References

[1] (2026). The Oaks | service details | North London NHS Foundation Trust. https://www.northlondonmentalhealth.nhs.uk/service-details/service/the-oaks-187/.

[2] (2026). Regional ethnic diversity - GOV.UK Ethnicity facts and figures. https://www.ethnicity-facts-figures.service.gov.uk/uk-population-by-ethnicity/national-and-regional-populations/regional-ethnic-diversity/latest/.

[3] (2026). How life has changed in the City of London: Census 2021. https://www.ons.gov.uk/visualisations/censusareachanges/E09000001.

[4] (2026). Our main sites - North London NHS Foundation Trust. https://www.northlondonmentalhealth.nhs.uk/our-main-sites.

[5] (2026). Sharing information with family members - ethical learning material - GMC. https://www.gmc-uk.org/professional-standards/learning-materials/sharing-information-with-family-members.

[6] Giacco D, Dirik A, Kaselionyte J, Priebe S (2017). How to make carer involvement in mental health inpatient units happen: a focus group study with patients, carers and clinicians. BMC Psychiatry.

[7] (2026). Dementia: assessment, management and support for people living with dementia and their carers. https://www.nice.org.uk/guidance/ng97.

[8] Hart JL, Turnbull AE, Oppenheim IM, Courtright KR (2020). Family-centered care during the COVID-19 era. J Pain Symptom Manage.

[9] (2026). Shared decision making. https://www.nice.org.uk/guidance/ng197/chapter/recommendations.

[10] Abou Seif N, Wood L, Morant N (2022). Invisible experts: a systematic review & thematic synthesis of informal carer experiences of inpatient mental health care. BMC Psychiatry.

[11] Wittenberg E, Kerr AM, Goldsmith J (2021). Exploring family caregiver communication difficulties and caregiver quality of life and anxiety. Am J Hosp Palliat Care.

[12] (2026). Living with COVID-19: visiting healthcare inpatient settings principles. https://www.england.nhs.uk/coronavirus/documents/c1606-living-with-covid-19-visiting-healthcare-inpatient-settings-principles/.

[13] (2026). Transition between inpatient mental health settings and community or care home settings. https://www.nice.org.uk/guidance/ng53/chapter/Recommendations.

[14] Azoulay E, Kentish-Barnes N (2020). A 5-point strategy for improved connection with relatives of critically ill patients with COVID-19. Lancet Respir Med.

[15] Langley GJ, Moen RD, Nolan KM, Nolan TW, Norman CL, Provost LP (1996). The Improvement Guide: A Practical Approach to Enhancing Organizational Performance, 2nd Edition. Jossey-Bass.

[16] Ogrinc G, Davies L, Goodman D, Batalden P, Davidoff F, Stevens D (2016). SQUIRE 2.0 (Standards for QUality Improvement Reporting Excellence): revised publication guidelines from a detailed consensus process. BMJ Qual Saf.

[17] (2026). Setting a SMART Aim | NLFT QI. https://qualityimprovement.northlondonmentalhealth.nhs.uk/qi-academy/resources-and-tools/setting-smart-aim.

